# Serial bone marrow transplantation reveals *in vivo *expression of the pCLPG retroviral vector

**DOI:** 10.1186/1743-422X-7-16

**Published:** 2010-01-22

**Authors:** Paula Fratini, Bryan E Strauss

**Affiliations:** 1 Setor de Vetores Virais, Laboratório de Genética e Cardiologia Molecular/LIM 13, Instituto do Coração, Faculdade de Medicina, Universidade de São Paulo, Av. Dr. Enéas de Carvalho Aguiar, 44, Bloco 2, 10 andar, São Paulo, SP, CEP 05403-900, Brasil; 2 Programa Interunidades em Biotecnologia, Instituto de Ciências Biomédicas, Universidade de São Paulo, São Paulo, CEP 05508-900, Brasil; 3 Instituto do Milênio- Rede de Terapia Gênica, Ministério da Ciência e Tecnologia, Esplanada dos Ministérios, Bloco E, Brasília, DF, CEP 70067-900, Brasil

## Abstract

**Background:**

Gene therapy in the hematopoietic system remains promising, though certain aspects of vector design, such as transcriptional control elements, continue to be studied. Our group has developed a retroviral vector where transgene expression is controlled by p53 with the intention of harnessing the dynamic and inducible nature of this tumor suppressor and transcription factor. We present here a test of *in vivo *expression provided by the p53-responsive vector, pCLPG. For this, we used a model of serial transplantation of transduced bone marrow cells.

**Results:**

We observed, by flow cytometry, that the eGFP transgene was expressed at higher levels when the pCLPG vector was used as compared to the parental pCL retrovirus, where expression is directed by the native MoMLV LTR. Expression from the pCLPG vector was longer lasting, but did decay along with each sequential transplant. The detection of eGFP-positive cells containing either vector was successful only in the bone marrow compartment and was not observed in peripheral blood, spleen or thymus.

**Conclusions:**

These findings indicate that the p53-responsive pCLPG retrovirus did offer expression *in vivo *and at a level that surpassed the non-modified, parental pCL vector. Our results indicate that the pCLPG platform may provide some advantages when applied in the hematopoietic system.

## Background

The merits and shortcomings related to the use of retroviral vectors for laboratory and clinical gene transfer have been intensely studied. Vectors derived from the Moloney Murine Leukemia Virus (MoMLV) hold an important, historical place in the development of clinical gene therapy. These vectors are relatively easy to produce and manipulate, are quite malleable and are extremely efficient, especially when applied *ex vivo *[1]. However, they have been associated with severe adverse events in clinical trials for the treatment of X-SCID [2] and the silencing of retroviral expression *in vivo *has been observed [3,4].

The MoMLV long terminal repeat (LTR) can be employed to drive transgene expression and is a robust promoter, especially in cultured cells. However, the viral promoter tends to suffer methylation and consequently is silenced, particularly when transduced hematopoietic stem cells (HSC) are transplanted in recipients [3,4]. Silencing of the MoMLV LTR can be avoided if the transgene contributes to positive selection of those cells that maintain viral expression [5]. In the X-SCID trials, the transgenes provided an advantage related to transduction of growth-promoting signals [6,7]. Many treatment protocols require the transfer of a therapeutic gene that does not contribute to positive selection. In this situation, prolonged vector expression may require modification of the LTR itself in order to promote transcription and avoid the cellular mechanisms that cause methylation [4].

In our previous studies, we altered the LTR of a typical MoMLV-derived vector such that transgene expression is driven by p53. This vector, called pCLPG, was shown to express reporter genes at levels superior to the parental vector, pCL, which utilizes the native MoMLV LTR to drive transgene expression [8]. We have also inserted the wild-type p53 cDNA under the control of this p53-responsive promoter and showed that an autoregulatory, positive feedback mechanism was established, resulting in improved expression of p53 as well as greater tumor cell inhibition when tested in tissue culture [9]. However, until now, we had not tested the pCLPG vector in a model that would test its potential for application *in vivo*.

Since retroviral vectors are best suited for *ex vivo *gene transfer and one of their typical uses in clinical trials has been in the hematopoietic system, we wished to test the pCLPG vector in such a model. Mouse models of serial transplantation of transduced bone marrow cells have often been used for this purpose since it places pressure on the stem cells to self renew and repopulate the hematopoietic system of the irradiated recipient [10,11]. In a relatively short period of time, this model can provide rigorous testing of the sustainability of vector expression. In addition, such models can also reveal potential adverse events related to the presence of the vector and transgene [12].

We show here that the pCLPG vector does indeed support expression *in vivo*. At least in the bone marrow compartment, expression from the pCLPG vector was sustained at a higher level and for a longer period of time than was seen for pCL. The use of a p53-responsive vector may prove to be an interesting option for gene transfer in the hematopoietic system.

## Results

### p53-responsiveness of the pCLPG vector in the context of a hematopoietic cell

**Figure 1:**
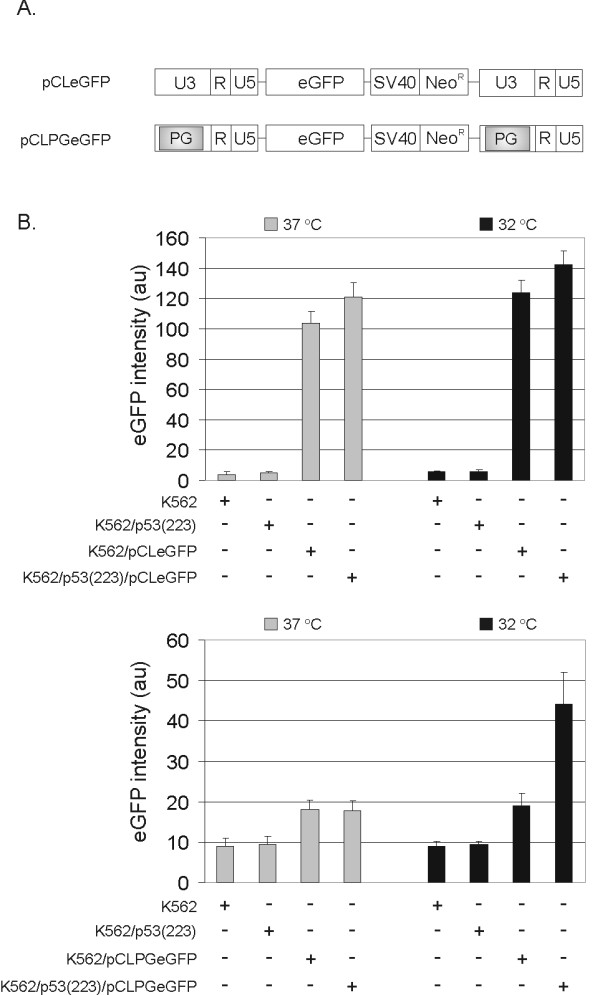
**p53-dependent expression from pCLPGeGFP revealed in a hematopoietic cell line**. (A) Schematic representation of the parental, non-modified gamma retroviral vector, pCLeGFP, which utilizes the native LTR to drive transgene expression. For pCLPGeGFP, the LTR was modified by the removal of the enhancer region and insertion of a p53-responsive element, as described previously [8,9]. (B) K562 cells were used to test expression of the pCLeGFP and pCLPGeGFP vectors in response to p53 activity (intensity of eGFP; au, arbitrary units). The data represent the mean and standard deviation of duplicate samples from 3 independent experiments.

A tissue culture assay was performed in order to determine if the expected p53-dependence of the pCLPG vector would be preserved in hematopoietic cells. For this, the pCLeGFP or pCLPGeGFP vectors (Figure 1A) were used to transduce K562 cells (human chronic myelogenous leukemia, p53-null) which were then selected for G418 resistance. The p53(223) temperature sensitive mutant [13] was introduced by a second round of retroviral transduction followed by selection with puromycin. The different cell types were then cultivated at either 32°C (permitting transactivational functions of the p53 mutant) or at 37°C (restricting p53 activity due to the mutant conformation of the protein). As shown in Figure 1B, the activation of the pCLPGeGFP vector was evident only when cells harboring this vector plus the p53(223) mutant were cultivated at 32°C. In contrast, the pCLeGFP vector was not affected by p53 status or temperature. This assay shows that, as expected, the pCLPG vector can be activated specifically by p53 in a hematopoietic cell.

### Serial transplantation of transduced bone marrow

**Figure 2:**
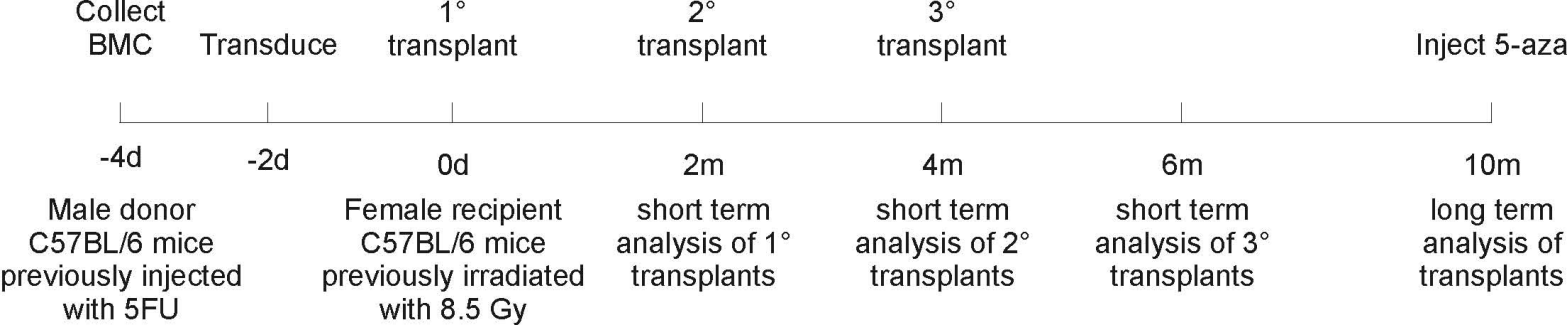
**Experimental design of serial transplantation**. Three groups of animals were treated with this procedure (mock-transduced BMC; animals receiving pCLeGFP-transduced BMC; animals receiving BMC transduced with pCLPGeGFP). Analysis included detection of eGFP by flow cytometry, hematologic exams, collection of gDNA and posterior determination of provirus copy number and chimerism.

In order to assess the expression of the p53-responsive pCLPG vector *in vivo*, a model of serial bone marrow transplantation was used. For this procedure, as shown in Figure 2, total bone marrow cells (BMC) were collected from male donor mice previously injected with 5-fluorouracil (5 FU). These cells were either mock transduced or transduced with the retroviral vectors pCLeGFP or pCLPGeGFP. These cells were then transplanted in isogenic female mice. The primary transplant recipients were maintained for two months before dividing the animals into sub-groups for analysis, continued observation or recovery of BMC for use in serial transplantation. In addition, male mice age-matched with the donor mice were maintained as controls for hematologic exams.

**Figure 3:**
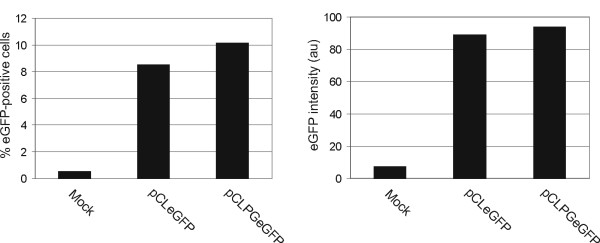
**Observation of eGFP expression in donor BMC**. Total BMC were collected from male donor mice, stimulated with cytokines and either mock transduced or transduced with pCLeGFP or pCLPGeGFP in the presence of Retronectin and analyzed by flow cytometry for eGFP expression 48-hours later (percentage of eGFP-positive cells and intensity of eGFP; au, arbitrary units).

The transduction of BMC with either the pCLeGFP or pCLPGeGFP vectors resulted in approximately 8 and 10% eGFP positive cells, respectively, as determined by flow cytometry (Figure 3). The intensity of eGFP fluorescence in cells transduced with either vector was quite similar, indicating that the expression from the p53-responsive pCLPG vector was as robust as the parental pCL virus. These cells were used to transplant the primary recipients, as described in Table 1, who were then used as donors in subsequent transplants.

**Table 1:** Summary of transplant procedure utilized

	1° Transplant	2° Transplant	3° Transplant
Survival of non-transplanted control animals	18-21d (n = 3)	18-22d (n = 3)	17-21d (n = 3)

Donor animals (male, C57BL/6)	n = 17	NA	NA

BMC collected	1-1.5 × 10^7 ^total cells/donor	1-1.5 × 10^7 ^total cells/donor	1-1.5 × 10^7 ^total cells/donor

Total number of recpients (female, C57BL/6)	n = 18	n = 17	n = 12

Donor animals used for serial transplant	n = 6^a^	n = 5^b^	NA

			

Short term maintenance of transplant recipients	64 d (n = 6)	59 d (n = 6)	62 d (n = 6)

			

Long term maintenance of transplant recipients	10 m (n = 6)^c^	8 m (n = 6)^c^	6 m (n = 6)^c^

a, Primary transplant recipients that were used as donors in the secondary transplant

b, Secondary transplant recipients that were used as donors in the tertiary transplant

c, Three animals in each long term group received 1 mg/kg 5-aza 24h prior to sacrifice

### Evaluation of eGFP expression in cells recovered from transplant recipients

**Figure 4:**
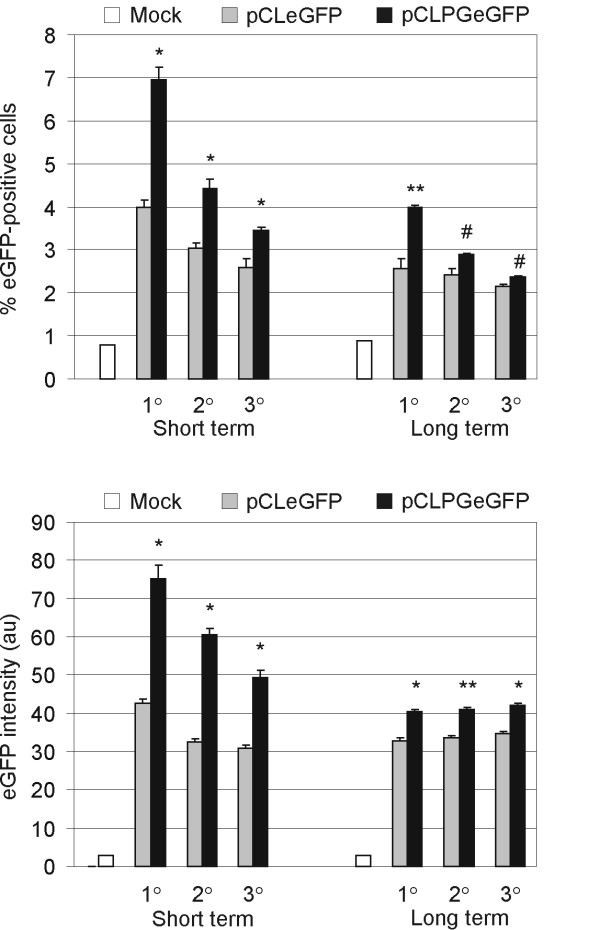
**Analysis of eGFP expression by flow cytometry in cells recovered from transplant recipients**. Short term (two months after transplant) or long term (6 to 10 months after transplant) observation cohorts were sacrificed after primar, secondary or tertiary transplantation (1°, 2° or 3°) and recovered cells were analyzed by flow cytometry for eGFP expression (percentage of eGFP-positive cells and intensity of eGFP; au, arbitrary units). Bars represent the mean and standard deviation among samples from the same cohort (please see Table 1 for the number of animals in each group). For statistical analysis, the Student's t-test (paired, 1-tailed; *, p < 0.0005; **, p < 0.005; #, p < 0.05) was performed using Excel, comparing pCLeGFP and pCLPGeGFP cohorts for each condition.

Analysis of eGFP expression in BMC, peripheral blood, spleen and thymus was performed after short or long term observation. eGFP-positive cells were observed only in BMC recovered from the transplant recipients, but not in the other tissues analyzed. As shown in Figure 4, the pCLPG vector provided superior expression of the transgene as compared to the parental pCL vector, especially among the primary and secondary recipients observed 2 months post-transplant. Expression from the pCLPG vector did decay by the tertiary transplant and in the animals maintained for long term observation. Though the level of pCLPG expression was significantly greater than that seen with the parental pCL vector, the difference was quite small, especially at the long term observation point.

Proviral copy number was assessed by real time PCR detection of vector sequences and comparing this level to a standard curve. As shown in Table 2, the number of provirus detected in the genomic DNA (gDNA) isolated from the BMC recovered from the transplanted animals ranged from 0.02-0.04 for the pCLeGFP group and 0.03-0.07 for the pCLPGeGFP group. We interpret this result as an indication that vector silencing was not observed in the BMC since the number of eGFP-positive cells closely matched the proportion of cells carrying provirus. However, the level of provirus was below the limit of quantification permitted by this assay when peripheral blood, spleen and thymus were analyzed (data not shown).

**Table 2:** Determination of provirus copy number and chimerism by real-time PCR

	Short term	
	**Chimerism^a^**	**Copy number (BMC)^b^**

**Non-transduced**	**ND**	**NA**

		

pCLeGFP		

1°	>95%	0.03-0.04

2°	>95%	0.02-0.03

3°	>95%	0.02-0.03

pCLPGeGFP		

1°	>95%	0.06-0.07

2°	>95%	0.04

3°	>95%	0.03

a, Chimerism determined using gDNA isolated from BMC recovered from transplant recipients where amplification of a Y-chromosome sequence was compared to a standard curve

b, copy number was determined by amplifying a viral sequence and comparing this to a standard curve. Note that provirus was not quantifiable by this assay in peripheral blood, spleen or thymus.

ND, not determined

NA, not applicable

Evaluation of chimerism was also performed by real time PCR detection of the Y chromosome in BMC recovered from the transplant recipients. In all cases, greater than 95% of the cells contained the Y chromosome, indicating that repopulation of the hematopoietic system in the female recipient mice was due to the implantation of the male BMC.

Hematologic exams were performed at the time of sacrifice of the transplanted animals. After short term observation, the only change that was noted was microcytic anemia (Table 3). This was present in the mock transduction as well as pCLeGFP and pCLPGeGFP groups, indicating that the presence of the vector was not responsible for this change. We show here only the tertiary transplant group since we would expect alterations, if they should occur, to be exaggerated in this group. The hematologic exams from the tertiary transplant recipients closely matched those of the other groups (Additional File 1). After long term observation, the apparent anemia was no longer present as evidenced by mean hemoglobin and mean corpuscular hemoglobin concentrations having returned to normal (Table 4 and Additional File 2).

**Table 3:** Representative hematologic analyses of transplant groups after short term observation

	Male age-matched mice	Non-transduced 3° transplant Short term	pCLeGFP 3° transplant Short term	pCLPGeGFP 3° transplant Short term
RBC× 10^6^/mm^3^	7.88 ± 0.10	8.51 ± 0.46	8.41 ± 0.42	8.51 ± 0.40

Hematocrit %	38.8 ± 1.16	46 ± 4.50	48 ± 4.0	46 ± 4.60

Hemoglobin g/dl	12.92 ± 0.34	11.92 ± 0.19	12.91 ± 0.34	12.92 ± 0.39

Mean globular volume % (MGV)	56.93 ± 3.02	60.83 ± 2.10	62.55 ± 3.50	60.64 ± 3.51

Mean hemoglobin concentration fl	30.21 ± 0.83	18.70 ± 3.68	19.92 ± 0.75	19.70 ± 0.61

Mean corpuscular hemoglobin concentration % (MCHC)	21.60 ± 1.80	36.16 ± 4.75	36.86 ± 4.90	37.16 ± 0.15

WBC× 10^3^/mm^3^	6.48 ± 0.14	4.27 ± 0.79	4.52 ± 0.98	4.27 ± 0.30

Eosinophils %	01 ± 0.75	02 ± 0.51	2.4 ± 0.75	02 ± 0.75

Monocytes %	02 ± 0.51	02 ± 1.36	03 ± 1.65	03 ± 0.89

Lymphocytes%	68.6 ± 2.68	67.6 ± 2.31	72.6 ± 0.98	77.5 ± 1.72

Neutrophils %	43.4 ± 2.09	28.75 ± 1.96	27.75 ± 2.25	29 ± 2.29

**Table 4:** Representative hematologic analyses of transplant groups after long term observation

	Male age-matched mice	Non-transduced 3° transplant Long term	pCLeGFP **3**° **transplant** Long term	pCLPGeGFP 3° transplant Long term
RBC× 10^6^/mm^3^	5.87 ± 0.034	6.31 ± 0.099	5.93 ± 0.39	6.18 ± 0.31

Hematocrit %	37.5 ± 1.29	35.6 ± 1.14	40.8 ± 2.39	42 ± 2.45

Hemoglobin g/dl	12.67 ± 0.027	11.57 ± 0.39	11.65 ± 0.43	11.77 ± 0.38

Mean globular volume % (MGV)	63.77 ± 0.099	65.066 ± .011	67.49 ± 0.47	67.92 ± 0.11

Mean hemoglobin concentration fl	21.25 ± 0.036	21.69 ± 0.23	22.64 ± 0.36	22.99 ± 0.15

Mean corpuscular hemoglobin concentration % (MCHC)	30.5 ± 2.38	32.49 ± 0.32	31.71 ± 0.45	32.12 ± 0.29

WBC× 10^3^/mm^3^	4.7 ± 0.059	4.62 ± .017	3.73 ± 0.26	4.024 ± 0.074

Eosinophils %	2 ± 0.82	1.4 ± 0.89	0.4 ± 0.55	0.4 ± 0.89

Monocytes %	3.75 ± 0.5	1.4 ± 0.54	1.2 ± 0.45	1.6 ± 0.55

Lymphocytes%	63.94 ± 0.077	68.45 ± 0.32	66.082 ± 0.46	65.92 ± 0.43

Neutrophils %	48 ± 1.14	44.76 ± 1.43	42.43 ± 0.51	42.85 ± 0.71

### *In vivo *treatment with 5-azacytidine corroborates lack of methylation in BMC

Animals from the long term observation groups were treated with 5-azacytidine (5-aza) *in vivo*, 24-hours prior to sacrifice. By flow cytometric evaluation of eGFP activity, we noted that little or no change was present in cells recovered from the bone marrow compartment, though peripheral blood, spleen and thymus each revealed an extremely modest increase in the number of eGFP-positive cells detected after 5-aza treatment (data not shown).

As an additional measure of the impact of 5-aza, quantification of proviral sequences by real time PCR was performed in samples recovered from both treated and control animals. When corrected for amplification of a genomic segment of the β-Actin gene, no alteration in provirus was observed (data not shown). Taken together, the lack of response to *in vivo *treatment with 5-aza may suggest that silencing of vector expression was not a significant issue in these assays.

## Discussion

Using a model of serial bone marrow transplantation, we have shown that expression from the p53-dependent pCLPG retroviral vector was superior to that of the parental, constitutive pCL vector. For both vectors, expression was limited to the bone marrow compartment and was not detected in peripheral blood, spleen or thymus. Since the number of eGFP-positive BMC was closely correlated with the provirus copy number detected in these cells, these results suggest that no vector silencing was observed in this compartment. In addition, *in vivo *treatment with 5-aza did not increase the number of eGFP-positive cells in the bone marrow compartment, corroborating the idea that silencing was not an issue in these cells.

Evaluation of provirus copy number present in the BMC recovered after the short term observation of primary transplant recipient mice suggests that 7/100 or 4/100 cells contained a single pCLPGeGFP or pCLeGFP provirus, respectively. We presume that only a single copy, on average, would have integrated in these cells due to the low overall transduction efficiency. Reports in the literature indicate that multiple copies would be present only at transduction efficiencies above 30% [14]. Moreover, eGFP expression in the BMC of the recipient mice was positive in approximately 7/100 or 4/100 cells recovered from the pCLPGeGFP or pCLeGFP transplant groups, respectively. This correlation between provirus copy number and observation of transgene expression suggests that vector silencing was not a problem, at least in BMC. The lack of vector expression in peripheral blood, thymus and spleen is also consistent with the difficulty in quantifying provirus in these tissues.

For our experiments, we chose to transduce total BMC since this procedure has long been, and continues to be, widely used. For example, classic studies from the group of Donald Kohn have shown methylation of the native MoMLV LTR, but reliable expression from a modified vector when total BMC was transduced and followed by serial transplantation [3,4,10,11,15]. The work of Andersson et al, 2003, also used total BMC for transduction with a GFP-expressing retrovirus which, upon transplantation, prevented rejection of eGFP-transgenic skin grafts [16]. In 2006, the group of Brian Sorrentino also used the tansduction of total BMC recovered from X-SCID mice followed by transplantation to reveal the phenotypic tendency of these cells to transform [17].

The *ex vivo *transduction procedure used here resulted in a relatively low level of gene transfer in bone marrow cells. A recent report indicates that retrovirus produced in NIH3T3-derived packaging cells offers some advantages for the transduction of hematopoietic stem cells, namely the production of fibronectin, yet 293T cells barely produce this protein [18]. The presence of fibronectin in the virus preparation facilitates the preloading of viral particles onto the culture dish. The use of Retronection (recombinant fibronectin fragment CH-296), intended to provide a substrate onto which the viral particles can be seeded, was not advantageous when virus was produced in 293T [18]. In addition, virus pseudotyped with the VSVg envelope is not efficiently preloaded on Retronectin [19]. Both of these findings are consistent with our previous study where virus was produced in 293T cells with either amphotropic or VSVg envelopes and transduction was performed either with or without Retronectin, resulting in little change to transduction efficiency in BMC or K562 [PF and BES, unpublished data]. The use of 293T cells for virus production may be partly to blame for the low transduction efficiency observed in our studies.

As revealed in the short term observation groups of our experiments, the expression of the p53-responsive pCLPG vector was maintained longer and at a higher level than was seen for the parental pCL vector, at least as revealed in the primary and secondary transplant recipients. Loss of pCLPG expression in BMC does not seem to be related to vector silencing, but instead to the gradual loss of transduced cells during successive transplant procedures. In contrast, viral expression in the long term observation groups was quite similar between the two vectors. This implies that the pCLPG, without additional activation of p53, is superior to the pCL vector, at least in BMC and during the first months following transplant.

Taken together, the use of total BMC and the low trasduction efficiency may have contributed to the low level of viral marking in peripheral blood, spleen and thymus. Since the true hematopoieitic stem cells represent only a small portion of BMC, the odds are low that these cells were transduced in our experiments. Though we did not characterize the transduced BMC prior to transplantation, it is possible that those cells which were transduced were of a more committed phenotype. This situation would also be consistent with the loss of viral marking upon successive transplants since the transduced cells would have a finite life span and would be expected to die over time.

Vector silencing by methylation is a common problem in retroviral vectors that use the non-modified MoMLV LTR to drive transgene expression. However, the pCLPG vector was modified in the LTR, prompting us to evaluate its performance in this serial transplantation model. If silencing by methylation had occurred, then treatment with 5-azacytidine should lead to an increase in eGFP-positive cells. Treatment with 5-aza did not result in the alteration of eGFP-positive cells in BMC. Therefore, direct assessment of methylation in the retroviral LTR by methylation-specific sequencing was not performed in this study.

Although further testing is still required, we propose that a p53-responsive vector may be beneficial for gene therapy applications in the hematologic system. For example, the expression of the splice-corrected MDR1 cDNA by pCLPG in HSC could be induced by chemotherapeutic drugs that activate p53, such as doxorubicin. In this scenario, the chemotherapy drug should not only kill tumor cells, but also induce the expression of MDR1 from the pCLPG vector and thus protect the hematopoietic system from the drug's effect, yet removal of the drug would result in the reduction in vector expression.

The insertion of the retroviral vector may induce the unwanted expression of a neighboring oncogene. In the case of pCLPG, the enhancer is dependent on p53 activity, implying that induction of the oncogene and p53 would be juxtaposed and may lead to elimination of these cells through apoptosis coordinated by p53. Lentiviral vectors are thought to be a safer alternative to gamma retroviral vectors [20]. The concept of p53-driven viral expression could be transferred to lentiviral vectors, maintaining the dynamic control over transgene expression and, possibly, gaining the relative safety of a vector that tends to integrate at a distance from gene promoters [21].

As revealed in the short term observation of primary and secondary transplant recipients, the pCLPG vector provided superior expression as compared to the parental vector. This indicates that gene transfer vectors that utilize p53 to drive transgene expression may be of interest for application in the hematopoietic system.

## Methods

### Viral vectors

The pCLeGFP and pCLPGeGFP vectors have been described previously [9,22]. It should be noted that in our previous work, the pCLPG vector was referred to as pCLPG-ΔU3, and has been re-named for simplicity.

### Virus production

To produce virus-containing supernatant, the indicated viral vectors were co-transfected in the 293T cells as described [23], except using pCMV-gag-pol and pCMV-VSVg packaging vectors (kindly provided by Richard Mulligan, Harvard Medical School, Boston, MA, USA and Jane Burns, University of California, San Diego, USA, respectively). After 24 hours of incubation, the virus-containing supernatant was collected, centrifuged for 5 minutes, 1000 ×g, then the supernatant removed and concentrated by ultracentrifugation (100,000 ×g, 120 min). The viral pellet was resuspended by overnight incubation in Hank's Balanced Salt then aliquoted and stored at -70°C.

### Titration of virus preparations

Titration was performed by transducing NIH3T3 cells then counting eGFP-positive cells by flow cytometry. This protocol has been described previously [22]. Typical titers were in the range of 1-3 × 10^6 ^green fluorescence units (gfu)/ml before ultracentrifugation and 2 × 10^8 ^gfu/ml after.

### Temperature sensitive p53 assay

K562 cells were transduced with pCLeGFP or pCLPGeGFP at an MOI of 1 then selected for G418 resistance. These cells were then transduced with a second retroviral vector, pLPCp53(223) (kindly provided by Andrei Gudkov, Lerner Research Institute, Cleveland, OH) which encodes the human p53 mutant P223L as well as the puromycin resistance gene. The cells were treated with puromycin, 1 μg/ml, until control cells had died. Approximately 1 × 10^6 ^cells of each type were plated in duplicate 6-well dishes. One dish was maintained at 37°C and the other at 32°C for 24 hours before harvesting the cells and analysis by flow cytometry of the percentage of eGFP-positive cells as well as the intensity of eGFP activity, as determined by the cytometry software.

### Collection and cultivation of bone marrow cells (BMC)

Animal handling procedures and experimental design was approved by institutional ethics committees (Biomedical Sciences Institute, protocol 097/04, as well as the School of Medicine, USP, research protocol SDS 2832/06/077). Young adult (approximately 90 days old), male C57BL/6 mice (obtained from the *Centro de Bioterismo*, FM-USP) were injected i.p. with 5-fluorouracil (5-FU), 150 mg/kg, and maintained for 7 days. The mice were then sacrificed and their tibias and femurs isolated. BMC were flushed from the bones upon washing with medium (Iscove's Modified Eagle Medium, IMDM, containing 15% fetal calf serum, FCS, Hyclone, USA). The BMC were centrifuged at 1000 ×g for 5 minutes and then resuspended in medium supplemented with recombinant mIL-3 (200 units/ml), hIL-6 (200 units/ml) and murine stem cell factor (mSCF, 2.5 ng/ml). Cytokines were obtained from Peprotech (Mexico). Cells were cultivated in a humidified 37°C, 5% CO_2 _incubator for 48-72 hours before continuing with the transduction.

### Transduction of BMC

Non-tissue culture treated 35 mm Petri dishes were treated with Retronectin (Takara, Japan), 20 μg/cm^2^, incubated with 1× phosphate buffered saline (PBS) containing 2% FCS for 30 minutes at 37°C, removed and then the treated plates pre-loaded with virus particles. For this, 2 × 10^7 ^virus particles were added and allowed to incubate at 37°C for 90 minutes, removed, and a fresh aliquot of virus was added for a second round of pre-loading. BMC cells, 4 × 10^6 ^(in medium plus cytokines) were then added to the dish along with a final aliquot of virus preparation, resulting in a multiplicity of infection (MOI) of 15. Cells were maintained during 48 hours before proceeding with transplantation.

### Transplantation of BMC

Recipient, isogenic young adult female animals were irradiated from a cobalt source, 8.5 Gy with attenuation (with professional assistance from Elisabeth Somesssari, Instituto de Pesquisas em Energia Nuclear, São Paulo). Immediately after irradiation, animals were injected i.v. with 4 × 10^6 ^BMC (with or without transduction, as indicated) in 100 μl of 1× PBS. In parallel, irradiated animals were maintained under the same conditions, but without having received a BMC injection, in order to serve as a control of the experimental procedure. Tetracycline, 100 mg/ml, was added to drinking water as a preventative measure to avoid infections and animals were maintained in micro-isolator cages until the controls had died, usually 18-21 days, and then the transplant recipients were maintained in standard cages.

### Hematologic analysis of peripheral blood

Blood was collected immediately upon sacrifice by cardiac puncture and mixed with EDTA to prevent coagulation. Differential counts were performed manually with Panotic stained blood smears. Assays were performed by the *Centro de Bioterismo*, FM-USP.

### Isolation of genomic DNA and detection of provirus and chimerism by Real-Time PCR

For the PCR reactions (performed in triplicate), 6.5 ng of gDNA, 200 nM of each primer, 10 μl of 2× SYBR Green PCR Master Mix (Applied Biosystems) and 7.4 μl of water were used. Control reactions without template or without primers were also performed. Amplification was carried out using a 7500 Fast System PCR (Applied Biosystems) under the following conditions: Stage 1, 95°C for 10 min; Stage 2, 40 cycles of 95°C for 15 sec and 60°C for 1 min; Stage 3: 95°C for 15 sec, 60°C for 1 min, 95°C for 15 sec followed by termination at 60°C for 1 min. The primers used were: pCLeGFP Forward (5' CCCGACAACCACTACCTGA-3') and pCLeGFP Reverse (5' TCCACACCCTAACTGACACA 3'), β-Actin Forward (5' AGAGGGAAATCGTGCGTGAC 3') and β-Actin Reverse (5' CAATAGTGATGACCTGGCCGT 3'), Y chromosome Forward (5' GCGCCCCATGAATGCAT 3') and Y chromosome Reverse (5' TCCACCTGCATCCCAGCT 3') with expected amplicons of 191, 137 and 112 base-pairs, respectively. The β-Actin and Y chromosome oligo design was derived from Mortellaro et al [24]. The pCLeGFP oligos (which also serve for detection of pCLPGeGFP) were designed using Primer 3 and Net Primer, then specificity was verified by BLAST. The β-Actin control served to ensure that variations were not due to errors in gDNA quantification and handling.

### *In vivo *treatment with 5-azacytidine

Animals maintained for long term observation (n = 6) in each group were subdivided. For each group, 3 animals were maintained as controls and the other 3 were injected i.p. with 1 mg/kg of 5-azacytidine (Sigma) in 1× PBS. After 24-hours, all animals were sacrificed and BMC, peripheral blood, thymus and spleen were collected for further analysis.

## Competing interests

The authors declare that they have no competing interests.

## Authors' contributions

PF contributed to the experimental design and carried out the experimental portion of this work. BES conceived of the study and drafted the manuscript. All authors read and approved the final manuscript.

## Supplementary Material

Additional file 1 **Table showing the complete hematologic exams (short term observation groups)**. Complete hematologic exams (short term observation groups). Click here for file

Additional file 2 **Table showing the complete hematologic exams (long term observation groups)**. Complete hematologic exams (long term observation groups). Click here for file
