# Supplementary material for: Serial bone marrow transplantation reveals in vivo expression of the pCLPG retroviral vector
Source: Virol J. 2010 Jan 22;7:16. doi: 10.1186/1743-422X-7-16 (PMC2845565; doi:10.1186/1743-422X-7-16)
Supplement: Additional file 1 — Table showing the complete hematologic exams (short term observation groups). Complete hematologic exams (short term observation groups). [file 1743-422X-7-16-S1.doc]

Additional File 1

Table 1a: Hematologic evaluation after short term observation of the age matched males.

|  | Standard reference^a^ | 2 months | 4 months | 6 months |
| --- | --- | --- | --- | --- |
| RBCx 10^6^/mm^3^ | **7.8±0.9** | 8.18±0.54 | 8.45±0.68 | 7.88±0.10 |
| Hematocrit % | **38.7 ± 6.0** | 42.2 ± 1.60 | 39.8 ± 2.73 | 38.8 ± 1.16 |
| Hemoglobin g/dl | **15.5 ± 4.9** | 13.9 ± 0.64 | 13.2 ± 0.87 | 12.92 ± 0.34 |
| Mean globular volume % (MGV) | **50.5 ± 5.7** | 44.82 ± 2.74 | 44.12 ± 2.39 | 56.93 ± 3.02 |
| Mean hemoglobin concentration fl | **34 ± 3.9** | 32.94 ± 0.82 | 38.47 ± 0.40 | 30.21 ± 0.83 |
| Mean corpuscular hemoglobin concentration%  (MCHC) | **17 ± 2.4** | 14.99 ± 1.06 | 15.69 ± 1.09 | 21.60 ± 1.80 |
| WBCx 10^3^/mm^3^ | **6.6 ± 1.6** | 4.42 ± 1.02 | 5.66 ± 0.67 | 6.48 ± 0.14 |
| Eosinophils % | **1.1 ±1.4** | 01 ± 0.81 | 0 ± 0 | 01 ± 0.75 |
| Monocytes % | **2.4 ±1.5** | 01 ± 0.75 | 01 ± 0.76 | 02 ± 0.51 |
| Lymphocytes% | **46 ±15** | 56.6 ± 2.48 | 67.46 ± 2.78 | 68.6 ± 2.68 |
| Neutrophils % | **50 ±24.9** | 41.8 ± 3.02 | 42.8 ± 3.60 | 43.4 ± 2.09 |

a, empirical values determined periodically by the animal facility

Additional File 1

Table 1b: Hematologic evaluation after short term observation of animals transplanted with non-transduced BMC.

|  | 1^o^ transplant | 2^o^ transplant | 3^o^ transplant |
| --- | --- | --- | --- |
| RBCx 10^6^/mm^3^ | 9.63 ± 0.79 | 6.59 ± 0.25 | 8.51 ± 0.46 |
| Hematocrit % | 32 ± 1.83 | 39 ± 2.73 | 46 ± 4.50 |
| Hemoglobin g/dl | 10.78 ± 0.43 | 10.78 ± 0.43 | 11.92 ± 0.19 |
| Mean globular volume % (MGV) | 37.83 ± 1.21 | 59.8 ± 2.58 | 60.83 ± 2.10 |
| Mean hemoglobin concentration fl | 12.56 ± 0.73 | 17.6 ± 0.63 | 18.70 ± 3.68 |
| Mean corpuscular hemoglobin concentration%  (MCHC) | 31 ± 1.03 | 33.70 ± 0.97 | 36.16 ± 4.75 |
| WBCx 10^3^/mm^3^ | 6.1 ± 0.41 | 3.5 ± 0.45 | 4.27 ± 0.79 |
| Eosinophils % | 04 ± 1.92 | 03 ± 0.75 | 02 ± 0.51 |
| Monocytes % | 07 ± 1.47 | 03 ± 1.50 | 02 ± 1.36 |
| Lymphocytes% | 53.6 ± 2.68 | 62.2 ± 3.79 | 67.6 ± 2.31 |
| Neutrophils % | 38.6 ± 3.72 | 30 ± 2.63 | 28.75 ± 1.96 |

Additional File 1

Table 1c: Hematologic evaluation after short term observation of animals transplanted with BMC transduced with pCLeGFP.

|  | 1^o^ transplant | 2^o^ transplant | 3^o^ transplant |
| --- | --- | --- | --- |
| RBCx 10^6^/mm^3^ | 9.55 ± 1.02 | 6.07 ± 0.24 | 8.41 ± 0.42 |
| Hematocrit % | 32.2 ± 2.3 | 40.2 ± 2.87 | 48 ± 4.0 |
| Hemoglobin g/dl | 11.22 ± 1.62 | 11.36 ± 0.74 | 12.91 ± 0.34 |
| Mean globular volume % (MGV) | 35.20 ± 1.32 | 48.95 ± 2.75 | 62.55 ± 3.50 |
| Mean hemoglobin concentration fl | 12.44 ± 0.92 | 17.59 ± 1.37 | 19.92 ± 0.75 |
| Mean corpuscular hemoglobin concentration%  (MCHC) | 29.87 ± 1.19 | 35 ± 0.59 | 36.86 ± 4.90 |
| WBCx 10^3^/mm^3^ | 4.6 ± 0.92 | 3.72 ± 0.56 | 4.52 ± 0.98 |
| Eosinophils % | 4.6 ± 1.09 | 2.8 ± 0.51 | 2.4 ± 0.75 |
| Monocytes % | 3 ± 0.98 | 1 ± 0.81 | 03 ± 1.65 |
| Lymphocytes% | 53.4 ± 3.80 | 69.6 ± 1.86 | 72.6 ± 0.98 |
| Neutrophils % | 38.9 ± 3.27 | 26 ± 2.04 | 27.75 ± 2.25 |

Additional File 1

Table 1d: Hematologic evaluation after short term observation of animals transplanted with BMC transduced with pCLPGeGFP.

|  | 1^o^ transplant | 2^o^ transplant | 3^o^ transplant |
| --- | --- | --- | --- |
| RBCx 10^6^/mm^3^ | 10.15 ± 1.30 | 6.08 ± 0.28 | 8.51 ± 0.40 |
| Hematocrit % | 35 ± 1.63 | 40.2 ± 3.20 | 46 ± 4.60 |
| Hemoglobin g/dl | 10.38 ± 1.47 | 11.36 ± 0.86 | 12.92 ± 0.39 |
| Mean globular volume % (MGV) | 36.28 ± 2.71 | 54.95 ± 2.92 | 60.64 ± 3.51 |
| Mean hemoglobin concentration fl | 10.59 ± 1.30 | 18.76 ± 0.86 | 19.70 ± 0.61 |
| Mean corpuscular hemoglobin concentration%  (MCHC) | 26.90 ± 2.70 | 35 ± 0.59 | 37.16 ± 0.15 |
| WBCx 10^3^/mm^3^ | 6.24 ± 0.83 | 3.72 ± 0.47 | 4.27 ± 0.30 |
| Eosinophils % | 03 ± 1.47 | 01 ± 0.51 | 02 ± 0.75 |
| Monocytes % | 3.8 ± 1.21 | 01 ± 0.81 | 03 ± 0.89 |
| Lymphocytes% | 57.8 ± 3.88 | 76.2 ± 2.22 | 77.5 ± 1.72 |
| Neutrophils % | 36.6 ± 3.48 | 26.85 ±1.26 | 29 ± 2.29 |
