# Supplementary material for: Serial bone marrow transplantation reveals in vivo expression of the pCLPG retroviral vector
Source: Virol J. 2010 Jan 22;7:16. doi: 10.1186/1743-422X-7-16 (PMC2845565; doi:10.1186/1743-422X-7-16)
Supplement: Additional file 2 — Table showing the complete hematologic exams (long term observation groups). Complete hematologic exams (long term observation groups). [file 1743-422X-7-16-S2.doc]

Additional File 2

Table 1a: Hematologic evaluation after long term observation of the age matched males.

|  | Standard reference^a^ | 10 months |
| --- | --- | --- |
| RBCx 10^6^/mm^3^ | **4.60±9.54** | 5.87±0.034 |
| Hematocrit % | **32.50±9.6** | 37.5±1.29 |
| Hemoglobin g/dl | **12.21±2.4** | 12.67±0.027 |
| Mean globular volume % (MGV) | **46.20±15.9** | 63.77±0.099 |
| Mean hemoglobin concentration fl | **14.77±9.8** | 21.25±0.036 |
| Mean corpuscular hemoglobin concentration%  (MCHC) | **31.28±4.92** | 30.5±2.38 |
| WBCx 10^3^/mm^3^ | **4.90±0.65** | 4.7±0.059 |
| Eosinophils % | **0.09±0.24** | 2±0.82 |
| Monocytes % | **1.38±4.08** | 3.75±0.5 |
| Lymphocytes% | **63.93±18.04** | 63.94±0.077 |
| Neutrophils % | **17.01±49.6** | 48±1.14 |

a, empirical values determined periodically by the animal facility

Additional File 2

Table 1b: Hematologic evaluation after long term observation of animals transplanted with non-transduced BMC.

|  | 1^o^ transplant | 2^o^ transplant | 3^o^ transplant |
| --- | --- | --- | --- |
| RBCx 10^6^/mm^3^ | 6.31±0.099 | 5.95±0.12 | 6.31±0.099 |
| Hematocrit % | 35.6±1014 | 38.8±1.09 | 35.6±1.14 |
| Hemoglobin g/dl | 11.57±0.39 | 11.59±0.29 | 11.57±0.39 |
| Mean globular volume % (MGV) | 65.066±0.12 | 65.68±0.32 | 65.066±.011 |
| Mean hemoglobin concentration fl | 21.69±0.23 | 22.23±0.21 | 21.69±0.23 |
| Mean corpuscular hemoglobin concentration%  (MCHC) | 32.47±0.32 | 31.44±0.29 | 32.49±0.32 |
| WBCx 10^3^/mm^3^ | 4.62±0.17 | 4.51±0.21 | 4.62±.017 |
| Eosinophils % | 1.4±0.89 | 0.8±.045 | 1.4±0.89 |
| Monocytes % | 1.4±0.54 | 1.2±0.45 | 1.4±0.54 |
| Lymphocytes% | 68.45±0.32 | 67.35±0.83 | 68.45±0.32 |
| Neutrophils % | 44.76±1.43 | 44.47±0.16 | 44.76±1.43 |

Additional File 2

Table 1c: Hematologic evaluation after long term observation of animals transplanted with BMC transduced with pCLeGFP.

|  | 1^o^ transplant | 2^o^ transplant | 3^o^ transplant |
| --- | --- | --- | --- |
| RBCx 10^6^/mm^3^ | 6.84±0.21 | 5.75±0.39 | 5.93±0.39 |
| Hematocrit % | 40.2±2.49 | 38.4±2.3 | 40.8±2.39 |
| Hemoglobin g/dl | 11.076±0.24 | 10.65±0.36 | 11.65±0.43 |
| Mean globular volume % (MGV) | 68.15±0.62 | 67.092±0.17 | 67.49±0.47 |
| Mean hemoglobin concentration fl | 22.36±0.35 | 22.91±0.18 | 22.64±0.36 |
| Mean corpuscular hemoglobin concentration%  (MCHC) | 34.34±0.5 | 32.26±0.45 | 31.71±0.45 |
| WBCx 10^3^/mm^3^ | 4.47±0.29 | 4.062±0.05 | 3.73±0.26 |
| Eosinophils % | 1.2±.0.44 | 0.8±0.45 | 0.4±0.55 |
| Monocytes % | 1.4±0.55 | 1.2±0.45 | 1.2±0.45 |
| Lymphocytes% | 68.38±0.56 | 65.86±0.36 | 66.082±0.46 |
| Neutrophils % | 44.18±0.75 | 43.47±0.31 | 42.43±0.51 |

Additional File 2

Table 1d: Hematologic evaluation after long term observation of animals transplanted with BMC transduced with pCLPGeGFP.

|  | 1^o^ transplant | 2^o^ transplant | 3^o^ transplant |
| --- | --- | --- | --- |
| RBCx 10^6^/mm^3^ | 6.92±0.1 | 5.89±0.36 | 6.18±0.31 |
| Hematocrit % | 41.8±2.49 | 40.2±1.79 | 42±2.45 |
| Hemoglobin g/dl | 11.36±0.76 | 11.038±0.69 | 11.77±0.38 |
| Mean globular volume % (MGV) | 68.51±0.41 | 67.51±0.46 | 67.92±0.11 |
| Mean hemoglobin concentration fl | 22.64±0.41 | 23.078±0.12 | 22.99±0.15 |
| Mean corpuscular hemoglobin concentration%  (MCHC) | 34.76±0.45 | 32.89±0.77 | 32.12±0.29 |
| WBCx 10^3^/mm^3^ | 4.82±0.16 | 4.23±0.094 | 4.024±0.074 |
| Eosinophils % | 1.2±0.45 | 0.6±0.55 | 0.4±0.89 |
| Monocytes % | 1±0 | 1.4±0.55 | 1.6±0.55 |
| Lymphocytes% | 68.74±0.45 | 66.39±0.42 | 65.92±0.43 |
| Neutrophils % | 44.43±0.79 | 43.46±0.27 | 42.85±0.71 |
